# Young women and breast cancer: challenges and answers—report from the Sixth Annual International Symposium, Mexico, 20–21 October 2014

**DOI:** 10.3332/ecancer.2014.495

**Published:** 2014-12-22

**Authors:** David Barros Sierra Cordera, Andrew Marx

**Affiliations:** 1 Fundación Mexicana para la Salud, AC, Periférico Sur No. 4809, Col El Arenal Tepepan, Delegación Tlalpan, 14610 México; 2 Harvard Global Equity Initiative (HGEI), 651 Huntington Avenue, François-Xavier Bagnoud Building, Room 632, Boston, MA 02115, USA

**Keywords:** breast cancer, young women, early detection, survivorship

## Abstract

Tómatelo a Pecho, Funsalud, the Harvard Global Equity Initiative, and the Mexican Ministry of Health led a group of institutions in organising the Sixth Annual International Symposium marking breast cancer awareness month in Mexico on 20–21 October 2014. This year’s event, with the theme ‘Young Women and Breast Cancer: Challenges and Answers’, took place at the National Institute of Perinatology in Mexico City.

This was the first time the symposium focused almost entirely on young women. The reasons for this emphasis were reported on by many national and global experts, who also presented evidence to show that breast cancer has become a leading cause of death among younger women in Mexico, and conveyed the benefits of early breast cancer detection and the need to create innovative solutions for care and survivorship support for this age group.

Over the course of one-and-a-half days, the symposium covered a wide range of topics and perspectives, including the epidemiology, biology, and genetics of breast cancer; challenges; and innovative answers to early detection and the myriad of short- and long-term challenges faced by patients with breast cancer, such as some cutting-edge techniques used to preserve fertility in women undergoing chemotherapy.

How the presence of local and global stakeholders will ensure the accountability of the multiple participants already immersed in the various areas of research and activities related to breast cancer. The voices of the Ministry of Health and of other institutions central to the Mexican health system show that there is a political will for work in this area, and there are the means to make a change happen.

Tómatelo a Pecho, Funsalud, the Harvard Global Equity Initiative (HGEI), and the Mexican Ministry of Health led a group of institutions in organising the Sixth Annual International Symposium marking breast cancer awareness month in Mexico on 20–21 of October 2014. This year’s event, with the theme ‘Young Women and Breast Cancer: Challenges and Answers’, took place at the National Institute of Perinatology in Mexico City.

Dr Felicia Knaul, President and Founder of Tómatelo a Pecho, Director of HGEI, and one of the leaders of the breast cancer awareness movement in Mexico, urged and inspired the audience to continue with their efforts to build evidence and develop innovative models of care for breast cancer, a disease that now kills 9.5 per 100,000 women in Mexico [1]. She was joined by Dr Mercedes Juan López, Mexico’s minister of health, who shared her vision of how Mexico can meet the challenges of breast cancer and officially inaugurated the symposium. She said ‘Breast cancer is no longer a synonym for death, but it is a synonym for struggle, the most important thing is to work on early and opportune detection, as well as adequate treatments which are fundamental for making this disease curable’.

Other speakers included Dr Julio Frenk, Dean of the Harvard TH Chan School of Public Health; Dr Abelardo Meneses García, Director General of the National Cancer Institute of Mexico; and Dr Rodrigo Medical, Director of the National Institute of Perinatology. These vastly experienced members of the panel highlighted the relevance of working among not only medical specialties but also across all sectors—and national boundaries—to bring the best of national and international strategies to local contexts. Intersectoral work is instrumental if global and local health systems are to reduce their huge burden, that reflected as 1.7 million new cases of breast cancer, and over 522,000 deaths in 2012, indeed one woman every minute [2].

This was the first time the symposium focused almost entirely on young women. The reasons for this emphasis were presented by many national and global experts, such as Dr Susan Love, who presented evidence that breast cancer has become a leading cause of death among younger women in Mexico, and conveyed the benefits of early breast cancer detection and the need to create innovative solutions for care and survivorship support for this age group. In Mexico, breast cancer is now the second leading cause of death for women between the ages of 30–54 [3]. In fact in low-income countries, as many as 66% of all women diagnosed with breast cancer are under 54 years of age, while in Canada, the United States, or Europe, it is merely 32% [4].

Over the course of one-and-a half days, the symposium covered a wide range of topics and perspectives, including: the epidemiology, biology, and genetics of breast cancer with epidemiologist Dr Rafael Lozano, who pointed out that breast cancer mortality in Mexico for women older than 25 years has increased by 35% since 1990, and we are seeing an increased mortality rate in the most marginalised communities [5].

The symposium also covered challenges and innovative answers to early detection through Dr Laura Magaña, with her experience in capacity-building in Mexico, and through Dr Ben Anderson’s experience as Chair of the Breast Cancer Global Initiative. Dr Anderson pointed out that ‘early detection only works when it’s followed by treatment’, and that this relationship depends on the strengthening of the health system itself.

But the symposium also placed an emphasis on breakthrough techniques developed to face short- and long-term challenges braved by patients with breast cancer. One of these is the need to preserve fertility in young women enduring chemotherapy. Dr Matteo Lambertini, mentioned that up to 80% of women older than 40 under chemotherapy and up to 20% of women younger than 30 years of age will develop premature ovarian failure [6].

Sharon Bober presented on the dire need for comprehensive understanding of and suitable care for the patient’s sexual health, since young women undergoing chemotherapy usually exhibit alterations to body image, self-esteem, premature menopause, and dyspareunia, among others.

The seminar concluded with remarks from Dr Cynthia Villarreal on some of the programmatic innovations developed for young women abroad, such as Dr Ellen Warner’s young women with breast cancer (PYNK) programme in Canada, and Erin Hasselberg’s participation in the ‘Young & Strong’ programme with the Dana-Farber Cancer Institute.

Dr Javier Davila Torres wrapped up the event by presenting some of the achievements made in Mexico and exposing some of the obstacles the Mexican health system will have to face in order to curb the impact of breast cancer.

## Conclusion

The concluding points were clear and are as follows: the need for a strong sensitisation campaign on the benefits of self and clinical examination and mammography to improve early detection and to help eradicate the myths and barriers around breast cancer care; the need and importance of genetic research in order to understand causality, and guarantee adequate treatment; the need for survivorship research in order to plan appropriate systemic measures; and other breast cancer specific research in the local arena.

The presence of local and global stakeholders will ensure the accountability of the multiple participants already immersed in the various areas of research and activities related to breast cancer. The voice of the minister of health and of other institutions central to the Mexican health system show that there is the political will to succeed in this area, and there is the means to make a change happen.
